# Leptomeningeal metastasis of lung adenocarcinoma presenting as cauda equina syndrome: a case report and literature review

**DOI:** 10.3389/fonc.2026.1955339

**Published:** 2026-09-28

**Authors:** Xiaofeng Chen, Haijuan Lv

**Affiliations:** Department of Radiology, the Second Affiliated Hospital of Jiaxing University, Jiaxing, China

**Keywords:** cast-like enhancement, cauda equina syndrome, leptomeningeal metastasis, lung adenocarcinoma, magnetic resonance imaging

## Abstract

**Objective:**

This study aims to improve the understanding of the imaging characteristics of leptomeningeal metastasis, particularly in patients presenting with cauda equina syndrome as the initial manifestation, in order to improve diagnostic accuracy and reduce the misdiagnosis rate.

**Methods:**

A 69-year-old male presented with a 4-month history of lower back pain accompanied by progressive lower limb numbness and gait instability. Non-contrast lumbar MRI performed at another hospital revealed punctate abnormal signal intensity in the cauda equina region, which was overlooked. The patient was treated as having lumbar disc herniation for 3 months without improvement. Chest CT subsequently revealed a left lower lobe pulmonary mass, which was surgically resected. Postoperative pathology confirmed poorly differentiated adenocarcinoma with neuroendocrine differentiation. Following surgery, the patient’s lower limb symptoms worsened, with an inability to walk and onset of urinary and fecal incontinence, prompting referral to our hospital. Physical examination revealed bilateral lower limb muscle strength of grade 2. Laboratory tests showed markedly elevated ferritin and mildly elevated NSE. Whole-spine and brain contrast-enhanced MRI were subsequently performed.

**Results:**

Spinal contrast-enhanced MRI demonstrated linear and nodular thickening and enhancement of the leptomeninges from the cervical to lumbar segments, dural thickening and enhancement, and soft tissue within the cauda equina region exhibiting cast-like filling with marked post-contrast enhancement. Brain contrast-enhanced MRI revealed multiple small nodular enhancing lesions. Based on the pathological confirmation of lung adenocarcinoma and the characteristic imaging findings, a probable (type II) diagnosis of extensive intracranial and leptomeningeal metastases was made on imaging grounds alone. The patient received systemic chemotherapy in combination with bevacizumab but had a poor response with rapid disease progression, and died one month after discharge.

**Conclusion:**

Cauda equina syndrome can be caused by leptomeningeal metastasis from malignant tumors, and the latter should be considered an important differential diagnosis for cauda equina syndrome in elderly patients. Contrast-enhanced MRI is essential for diagnosis, commonly revealing linear or nodular leptomeningeal enhancement, with cast-like thickening and enhancement representing a characteristic manifestation of advanced disease.

## Introduction

Leptomeningeal metastasis is a rare but serious complication of advanced malignancies, typically progressing rapidly with a poor prognosis, yet diagnosis is often delayed. Lung cancer is the leading cause of central nervous system (CNS) metastases in adults (1). Leptomeningeal metastasis represents a relatively rare subtype, accounting for only 3.8% of CNS metastases in lung cancer patients (2). When the cauda equina roots are involved, the condition may manifest as cauda equina syndrome (CES), which is prone to misdiagnosis as lumbar disc herniation, thereby delaying appropriate treatment. Herein, we report a case of extensive CNS metastasis from lung adenocarcinoma presenting with CES as the initial manifestation, with particular attention to the MRI features of lumbosacral leptomeningeal metastasis.

## Case presentation

A 69-year-old male presented with a chief complaint of lower back pain accompanied by progressive lower limb numbness and gait instability of 4 months’ duration. He denied cough, sputum production, hemoptysis, dyspnea, appetite loss, unintentional weight loss, fever, or other constitutional symptoms. Non-contrast lumbar MRI performed at the initial hospital showed punctate abnormal signal intensity in the cauda equina region (**Figures 1A, B**), which was overlooked. The patient was misdiagnosed with lumbar disc herniation and treated accordingly for 3 months without improvement; the specific treatment details were not available, and he was subsequently admitted to the neurology department of a local hospital one month prior. Chest CT on admission revealed a left lower lobe pulmonary mass (**Supplementary Figures 1A, B**), which was surgically resected. Histopathological examination of the left lower lobe pulmonary mass revealed a poorly differentiated adenocarcinoma with neuroendocrine differentiation. Immunohistochemical staining showed positivity for TTF-1 and CK7, while Napsin A and P40 were negative, supporting pulmonary adenocarcinoma differentiation. The tumor cells were also positive for the neuroendocrine markers, including synaptophysin (Syn), CD56, and chromogranin A (CgA), indicating neuroendocrine differentiation. EGFR showed focal positivity, whereas ALK, CD10, D2-40, CD34, and p53 were negative. The Ki-67 proliferation index was approximately 30%. Molecular testing identified a KRAS G12V mutation. After surgery, the patient’s lower limb numbness and pain worsened, with inability to walk, sharp buttock pain, and onset of urinary and fecal incontinence, prompting referral to our hospital.

**Figure 1:**
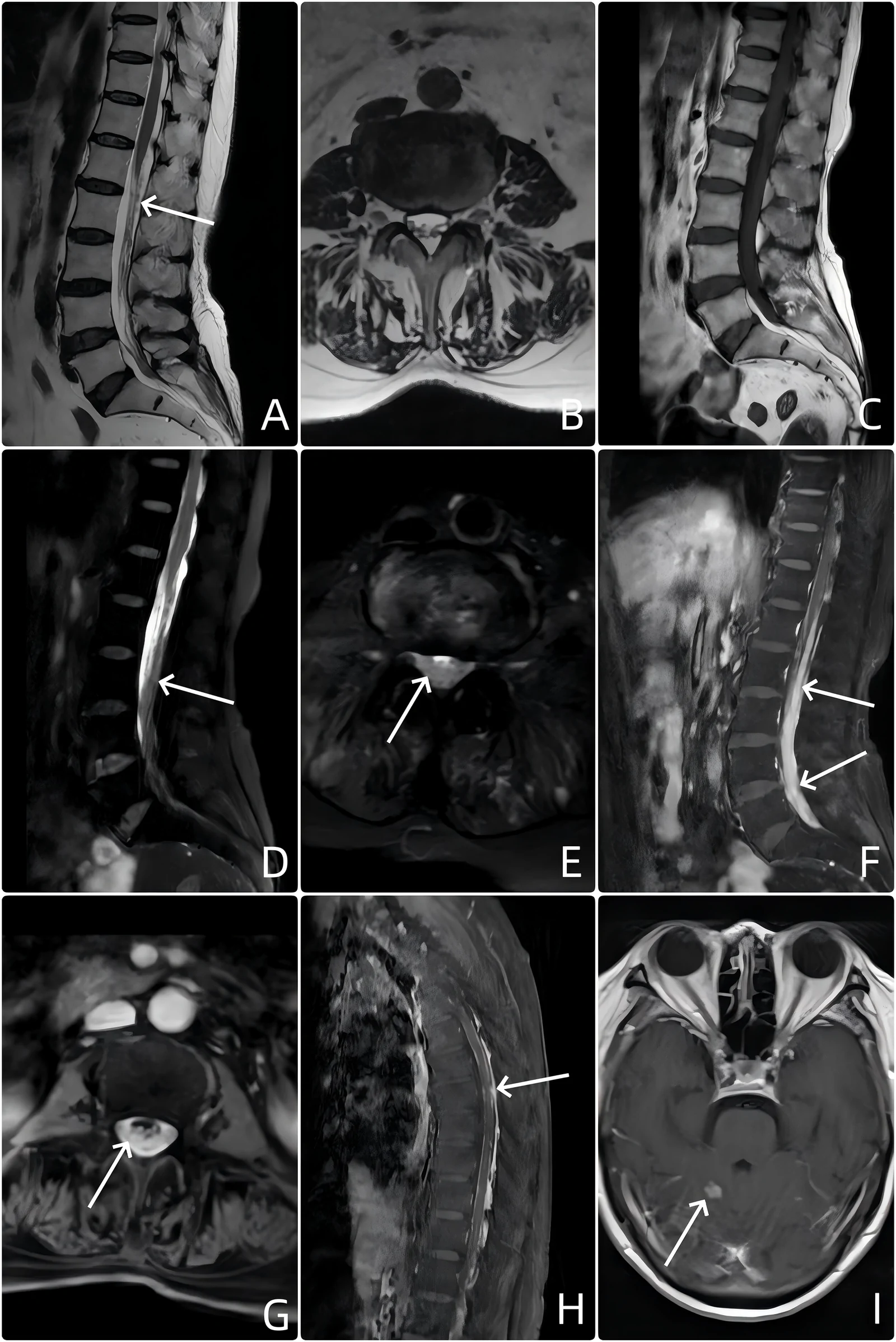
Imaging findings of leptomeningeal metastasis from lung adenocarcinoma. **(A, B)** Non-contrast lumbar MRI performed 3 months earlier at another hospital: sagittal T2WI demonstrates multiple small nodular isointense lesions on the surface of the cauda equina **(A)**; axial T2WI at the L3/4 disc level shows poorly visualized lesions **(B)**. **(C)** Sagittal T1WI of the lumbar spine during current hospitalization: no definite abnormal signal is observed within the lumbar thecal sac. **(D)** Fat-suppressed sagittal T2WI demonstrates strip-like iso- to hypointense lesions within the thecal sac relative to CSF. **(E)** Axial T2WI at the same level shows the lesion occupying the majority of thecal sac space, with poorly visualized cauda equina. **(F)** Fat-suppressed contrast-enhanced sagittal T1WI clearly demonstrates “cast-like” enhancing lesions within the lumbosacral thecal sac. **(G)** Fat-suppressed contrast-enhanced axial T1WI shows marked enhancement of the intradural lesions, with the cauda equina encased and poorly visualized. **(H)** Coronal contrast-enhanced thoracic MRI demonstrates the striking contrast between the hypointense spinal cord and the linear/nodular enhancing tumor implants, exhibiting the “sugar-coating sign.” **(I)** Brain contrast-enhanced MRI shows an enhancing nodule in the right cerebellum.

## Laboratory and physical examination

On admission, vital signs were stable. Muscle strength was grade 5/5 in both upper extremities and grade 2/5 in both lower extremities. Deep tendon reflexes in the lower extremities were diminished bilaterally. Sensory examination revealed numbness in the saddle region. The right Babinski sign was equivocal, and the left Babinski sign was negative. Laboratory tests showed CEA 1.66 ng/mL, NSE 8.7 ng/mL, ferritin 987.4 ng/mL, white blood cell count 8.33 × 10^9^/L, and CRP 1.33 mg/L.

## CT and MRI examination

During the current hospitalization, contrast-enhanced lumbar MRI revealed that the lumbosacral thecal sac was filled with lesions appearing hypointense on T1-weighted imaging (T1WI) and isointense on T2-weighted imaging (T2WI), with marked homogeneous enhancement post-contrast. The cauda equina nerve roots were encased and poorly visualized (**Figures 1C–G**). Compared with the MRI performed 3 months earlier at another hospital, the lesions had progressed significantly. Concurrently, thoracic contrast-enhanced MRI in the sagittal plane demonstrated a striking contrast between the hypointense spinal cord and the linear and nodular enhancing tumor implants within the thecal sac, exhibiting the characteristic “sugarcoating sign” (3) (**Figure 1H**); these findings were not clearly visible on non-contrast T1WI or T2WI. Brain MRI revealed nodular abnormal enhancement in the right cerebellum (**Figure 1I**). Lumbar puncture for CSF examination was not performed because the patient’s family refused the procedure in light of the patient’s poor performance status and rapid clinical deterioration. The EANO-ESMO guidelines recommend contrast-enhanced MRI of the brain and spinal axis for suspected leptomeningeal metastasis, supplemented by CSF evaluation as needed (4). Since the diagnosis was based on clinical presentation and contrast-enhanced MRI findings alone, without cytological or biochemical CSF confirmation, the case was classified as probable (type II) leptomeningeal metastasis according to the EANO-ESMO diagnostic criteria (4).

## Treatment and follow-up

As no G12V-selective inhibitor was available, the patient was treated with systemic chemotherapy combined with anti-angiogenic therapy. Beginning July 24, 2024, he received bevacizumab 500 mg, nanoparticle albumin-bound paclitaxel 300 mg, and carboplatin 450 mg, all on day 1, administered every 3 weeks. However, the treatment was ineffective, the patient’s condition deteriorated rapidly with progressive disturbance of consciousness, and he died one month later.

## Discussion

We report a case of leptomeningeal metastasis from lung adenocarcinoma presenting with CES as the initial manifestation, with rapid progression over 3 months. Diffuse cast-like tumor growth filling the lumbosacral thecal sac may represent a characteristic manifestation of advanced leptomeningeal metastasis.

Cauda equina syndrome is a constellation of symptoms caused by compressive or traction injury to the cauda equina nerve roots, resulting in rectal and bladder dysfunction, saddle anesthesia, and diminished or absent lower limb sensory and motor function. Its classic presentation includes urinary retention, fecal or urinary incontinence, sensory deficits in the saddle distribution corresponding to the affected nerve roots, back pain, and sexual dysfunction (5). The most common etiologies of CES include compression, infection, or congenital abnormalities, with malignant metastasis accounting for approximately 1%–8% of cases (6). CES caused by advanced-stage malignancies is rare but highly debilitating. A systematic review of intradural cauda equina metastases identified lung (18.7%), breast (13%), and renal (11.4%) carcinomas as the most frequent primary tumors (7). A review of previously reported cases of lung cancer–associated cauda equina syndrome (**Table 1**) reveals marked histological heterogeneity, encompassing adenocarcinoma, small cell lung carcinoma, squamous cell carcinoma, large cell neuroendocrine carcinoma, and poorly differentiated carcinoma with neuroendocrine differentiation; adenocarcinoma and small cell carcinoma are the most frequent.

**Table 1:** Reported cases of cauda equina syndrome caused by lung cancer.

Study	Gender/age	Lung cancer histology	Imaging findings	Treatment	Outcome
Pagano (8)	M/63 y	SCLC	multiple nodular small lesions on the entire cauda equina with contrast-enhancement	Partial tumor resection + palliative radiotherapy (stereotactic radiosurgery)	Death 6 months later
Nicoletti (9)	F/55 y	AC	Lumbar MRI misread as schwannoma	Subtotal tumor resection + chemotherapy + radiotherapy	Postoperative status at 1 year: acceptable
Liu (10)	M/59 y	AC	MRI: multiple nodules at conus medullaris/cauda equina	chemotherapy	Posterior cranial hypertension developed after 1 month; death occurred after 3 months
Xiong (11)	M/55 y	SCLC	MRI: solitary well-demarcated intradural extramedullary mass at L3/4	Tumor resection + chemotherapy	Postoperative cauda equina symptoms improved rapidly; the patient died one year later
Cansever (12)	M/79 y	SCC	MRI: intradural mass (13×11 mm) at L2–3 infiltrating the cauda equina	Total tumor resection	Mild improvement in neurological function; transfer to another hospital
Lin (13)	M/79 y	SCLC	L2–L3 tumor	Subtotal tumor resection	Death 1 month postoperatively
Kotil (14)	M/50 y	AC	Lumbosacral MRI: well-demarcated intradural extramedullary mass at L4/5	Total tumor resection + chemotherapy + radiotherapy	Postoperative remission; no recurrence observed at 2-year follow-up
Tsimpas (15)	M/62 y	LCNC	heterogeneously enhancing intradural extramedullary mass at L2/L3	Partial tumor resection + radiotherapy	Back pain improves, but foot function is lost.
Sakai (16)	M/40 y	AC	Multiple small nodules on the spinal cord surface and cauda equina	Gefitinib	Neurological symptoms resolved within 2 weeks; no recurrence was observed during the 4-month follow-up period

M, male; F, female; y, years; AC, Adenocarcinoma; SCC, Squamous cell carcinoma; LCNC, large-cell neuroendocrine carcinoma; SCLC, small‐cell lung carcinoma.

Most cases reported in the literature involved solitary nodules or masses at the L2–L5 level (9, 11–13, 15), some of which mimicked schwannomas on imaging. In contrast, our case demonstrated more extensive metastatic involvement, suggesting that the tumor may have disseminated longitudinally along the leptomeningeal and cerebrospinal fluid pathways.

Previous literature has reported that leptomeningeal metastasis typically manifests as diffuse linear leptomeningeal enhancement (17), also descriptively termed the “sugarcoating sign” in imaging literature (3), while cauda equina metastases may present as small nodules or localized masses on the cauda equina roots (10). The cervical and thoracic MRI findings, as well as the early-stage lumbar MRI findings, in the present case are consistent with the literature. However, serial imaging showed substantial progression of the lumbosacral lesions. Initially, only subtle punctate abnormalities were visible in the cauda equina region on non-contrast MRI. At the time of referral, contrast-enhanced MRI demonstrated confluent intrathecal enhancing tissue occupying much of the lumbosacral thecal sac, with encasement and poor visualization of the cauda equina roots. This morphology appeared to mold to the contour of the thecal sac and is described descriptively in this report as “cast-like intrathecal enhancement”, to our knowledge, this cast-like intrathecal filling pattern has not been reported in the literature; a PubMed search (6 September 2026) for ‘cast-like’ cauda equina leptomeningeal metastasis found no such description. This observed imaging appearance may arise from the progressive confluence of initially discrete linear and nodular leptomeningeal implants. As the tumor burden increased, the lesions produced the appearance of confluent intrathecal filling. The distribution of leptomeningeal tumor cells within the spinal subarachnoid space is influenced by cerebrospinal fluid flow and the seeding of tumor along the leptomeninges (17).

Cast-like metastasis within the lumbosacral thecal sac is rare, and its imaging appearance is not yet well recognized by the majority of radiologists, making it susceptible to misdiagnosis or missed diagnosis. On imaging, lumbosacral intraspinal leptomeningeal metastatic lesions must be differentiated from other intradural extramedullary tumors, including schwannoma, meningioma, ependymoma, and lymphoma. A solitary, well-circumscribed nodule or mass favors the possibility of a primary extramedullary tumor (18, 19). Diffuse nerve root or leptomeningeal enhancement should also be differentiated from inflammatory or infectious conditions such as arachnoiditis, radiculitis, tuberculous/fungal meningitis, and Guillain-Barré syndrome, which generally lack discrete mass formation and require comprehensive assessment incorporating CSF analysis and clinical evidence of infection. In addition, rare intraspinal space-occupying lesions such as intradural disc herniation, histiocytosis, and vascular lesions should be considered in the differential diagnosis in select cases (20, 21).

A key limitation of this report is that CSF examination was not available; accordingly, our diagnosis is classified as probable (type II) rather than confirmed (type I) LM under the EANO-ESMO framework, and definitive confirmation would require CSF cytology and/or biochemical analysis.

## Conclusion

For elderly patients presenting with unexplained low back and leg pain, progressive lower limb numbness, and bowel/bladder dysfunction, spine MRI findings should be interpreted with caution. When leptomeningeal metastasis is suspected, contrast-enhanced spinal MRI should be performed as early as possible; however, a definitive diagnosis of leptomeningeal metastasis also requires systemic imaging (e.g., CT or PET/CT), when clinically indicated, to identify the primary malignancy, together with cerebrospinal fluid (CSF) cytology when feasible. Cast-like enhancement of the thecal sac should be regarded as a descriptive imaging pattern, which may represent a characteristic imaging manifestation of advanced leptomeningeal metastasis, indicating extensive tumor seeding and a poor prognosis.

## Data Availability

The original contributions presented in the study are included in the article/**Supplementary Material**. Further inquiries can be directed to the corresponding author.

## Supplementary material

Supplementary Figure 1 **(A, B)** Chest CT of the primary tumor. A mass in the superior segment of the left lower lobe, with partially irregular margins and an internal air-bubble sign.
